# Metabolomic profiles of the silage digested in ruminal fermentation differentiated by various solvent extraction

**DOI:** 10.5455/javar.2025.l923

**Published:** 2025-06-02

**Authors:** Ayu Septi Anggraeni, Anuraga Jayanegara, Anjar Windarsih, Suratno, Ahmad Sofyan, Erika Budiarti Laconi, Nur Rochmah Kumalasari

**Affiliations:** 1 Research Center for Food Technology and Processing, National Research and Innovation Agency, DI Yogyakarta, Indonesia; 2 Department of Animal Nutrition and Feed Technology, Faculty of Animal Science, IPB University, Bogor, Indonesia; 3 Research Center for Animal Husbandry, National Research and Innovation Agency, Bogor, Indonesia

**Keywords:** Chitosan, metabolome, rumen fluid, sample differentiation, solvent, TMR silage

## Abstract

**Objective:**

The aim of this study was to examine the solvent conditions suitable for preparing samples of total mixed ration (TMR) silage and ruminal fluid, with or without chitosan inclusion, to achieve more sensitive, specific, and repeatable analyses.

**Material and Methods:**

The metabolome contained in the sample was determined using a ultra-high-performance liquid chromatography-high-resolution mass spectrometry quadrupole using samples in the form of TMR silage (silage with 0.5% inclusion of chitosan, silage without chitosan addition) and rumen fluid (rumen fluid containing 0.5% chitosan, ruminal fluid without chitosan) with and without the addition of chitosan, as well as three different solvent ratios: 50% water MS grade: 50% methanol (MeOH) MS grade (1), 20% water MS grade: 80% MeOH MS grade (2), and 0% water MS grade: 100% MeOH MS grade (3).

**Results:**

The TMR silage samples contained 311 metabolites, amino acid compounds predominating, followed by fatty acids and amines. Fatty acids, organic phosphate, and amines dominated the 39 metabolites found in rumen fluid samples. In the TMR silages, 100% MeOH seemed to be able to distinguish samples more effectively than other solvents; moreover, cinnamic acid, D-(+)-proline, and L(+)-ornithine were the three highest variable importance for projection values among prospective metabolite markers for silage samples. Whereas the use of a 50% MeOH to 50% water solvent ratio achieved the best discrimination results in rumen fluid samples, the three highest variable importance for projection values among prospective metabolite markers for ruminal fluid samples were triethyl phosphate, dibenzylamine, and phosphoric acid.

**Conclusion:**

100% MeOH is able to distinguish TMR silage, while 50% MeOH to 50% water is best for ruminal fluid samples.

## Introduction

The emerging field of metabolomics, a branch of omics methodology, focuses on understanding the alterations in metabolites triggered by external stimuli or disruptions [1]. Untargeted metabolomics, a comprehensive screening technique for evaluating metabolite compositions in specific samples, has gained prominence in recent [2]. In the realm of metabolic profiling, nuclear magnetic resonance spectroscopy, gas chromatography-mass spectrometry, and liquid chromatography-mass spectrometry stand out as widely used analytical platforms [3]. Because of its outstanding sensitivity, selectivity, and precision in detecting natural metabolites, untargeted metabolomics by means of liquid chromatography-high resolution mass spectrometry (LC-HRMS) has been implemented frequently. However, this approach requires a comprehensive understanding of the chemicals that influence fundamental biological activities, considering the variations in solvent and extraction procedures [4].

In the context of silage and rumen fluid samples, LC-HRMS has been instrumental in fully screening metabolites with small molecular sizes, offering broad coverage and high sensitivity in MS/MS detection [2,5]. Notably, metabolomics has significantly contributed to the exploration of silage [6–8] and ruminal [9,10], revealing novel and previously unidentified compounds. These insights suggest that silage and ruminal metabolome profiling can raise our understanding of the molecular mechanisms that underlie silage and ruminal fluid production [7]. Conversely, chitosan, an antibacterial organic compound, has demonstrated its capability to modulate the rumen microbial population [11]. Extensive research has shown that chitosan positively influences feed consumption, digestion, fermentation, enteric methane production, and silage quality [12].

The effectiveness and reproducibility of metabolite extraction and sample preparation strategies play a critical role in metabolomics investigations, affecting the characteristics of detected metabolites and subsequent biological interpretations. The choice of solvents, whether polar or non-polar, should aim to be simple, rapid, and extract as many metabolite classes as possible [13,14]. Various solvents and extraction techniques have produced different results when used for metabolite extraction. No single extraction method is capable of isolating all metabolites with equal efficiency while simultaneously fulfilling the demands for high throughput and practical handling [13]. The exploration of extraction solvents and methods is crucial to obtain the highest yield of bioactive metabolites from samples [4]. Polarity-indexed solvents, aligned with the desired polarities of the target metabolites, are commonly used for broad-spectrum extraction [15]. Various studies have demonstrated the impact of solvents, such as methanol (MeOH), acetone, and acetonitrile, on noncovalent bonds between proteins and other components in biofluids, influencing metabolite extraction outcomes [16]. In addition, a variety of organic solvents, including varying amounts of MeOH and water, have been used for metabolite extraction [3]. MeOH precipitation has proven to be a successful, simple, and reproducible method, with high protein removal efficiency (98%) and potential benefits for chromatographic column longevity and electrospray interface contamination reduction [5].

Metabolomics has been widely applied to the study of silage and ruminal fluids; however, there is a significant gap in understanding the metabolite composition of total mixed ration (TMR) silages and ruminal fluids treated with chitosan. Specifically, the optimal solvent extraction methods for such samples remain unexplored, as does the ability of these methods to distinguish metabolites between chitosan-treated and untreated samples. This seeks to address these gaps by systematically investigating and comparing metabolite profiles of TMR silages and ruminal fluids with and without chitosan treatment, utilizing various solvent extraction techniques. By optimizing solvent conditions, the research will identify treatment-specific metabolites, offering new insights into chitosan’s influence on metabolomic composition and advancing sample preparation methods in metabolomics. The objective is to assess the effects of different solvent extraction strategies on metabolite yield, profiling, and diversity in TMR silages and ruminal fluids treated with chitosan.

## Materials and Methods

### Ethical approval

This study was approved by the Ethical Clearance Committee of the Integrated Research and Testing Laboratory for Preclinical Experiments, Universitas Gadjah Mada, Yogyakarta, Indonesia, under Approval Number: 00004/04/LPPT/IV/2022.

### Silage extraction for metabolomics analysis

Silage was subjected to non-targeted metabolite profiling based on Guan et al. [7] and Windarsih et al. [2]. Ten grams of fresh silage are processed using the freeze-drying method, ground and then placed on a 1.5 ml microcentrifuge tube with 100 mg of silage sample. For silage without chitosan addition (SM) and silage with 0.5% inclusion of chitosan (SC), samples were extracted using three different types of solvent: 50% water MS grade: 50% MeOH MS grade (1), 20% water MS grade: 80% MeOH MS grade (2), and 0% water MS grade: 100% MeOH MS grade (3). The total volume for each solvent was 1 ml. The sample and solvent were sonicated for 30 min at room temperature after being vortexed for 30 sec. The samples were centrifuged for 10 min at 4°C at 12,000 rpm to separate the supernatant from the pellet. A 0.22 m PTFE filter and a 10 ml syringe were utilized for collecting the supernatant. For LC-HRMS analysis, the supernatant was injected. As a blank for metabolomic analysis, MS-grade MeOH and water were produced in the same proportion as the treatment sample.

### Ruminal fluid extraction for metabolomics analysis

Ruminal fluid was subjected to non-targeted metabolite profiling in accordance with Artegoitia et al. [9] and Windarsih et al. [2]. Based on the *in vitro* gas test procedure, rumen fluid was collected. Two crossbred Ongole fistula cattle that were previously kept on a diet of pasture (*Pennisetum hybrid*) and concentrate (60:40 on dry matter) were used to provide rumen fluid for *in vitro* examination. Rumen fluid was collected using the technique by Anggraeni et al. [17], in which 200 mg of rumen fluid was sampled and placed in a 1.5 ml microcentrifuge tube.

Metabolomic analysis was performed by using LC-HRMS. Samples were extracted with three different types of solvents for rumen fluid without chitosan addition (RM) and rumen fluid containing 0.5% chitosan (RSC). MS grade (1) consisted of 50% water and 50% MeOH; MS grade (2) contained 20% water and 80% MeOH, while MS grade (3) was composed entirely of 100% MeOH with no water content. The total volume of each solvent used was 1 ml. The sample and solvent were sonicated for 30 min after vortexing at ambient temperature for 30 sec. After that, samples were centrifuged for 10 min at 4°C at 12,000 rpm to separate the supernatant from the pellet. A 0.22 m PTFE filter and a 10 ml syringe were utilized to collect the supernatant. For LC-HRMS analysis, the supernatant was prepared to be injected. MS-grade MeOH and water were mixed in the same ratio as the treatment sample as a blank for metabolomics analysis.

### Metabolomics analysis using LC-HRMS analysis

Metabolic analysis was performed based on the methodology of Windarsih et al. [2]. Thermo Scientific’s Vanquish UHPLC Binary Pump and Q Exactive Hybrid Quadrupole-Orbitrap High-Resolution Mass Spectrometer for liquid chromatography and Orbitrap high-resolution mass spectrometry, respectively, were utilized for the analysis. Thermo Scientific™ Accucore™ 100 mm × 2.1 mm ID, 2.6 m Phenyl-Hexyl analytical column was used for liquid chromatography. With a gradient method and a flow rate of 0.3 ml/min, the mobile phases utilized were MS-grade water containing 0.1% formic acid (A) and MS-grade acetonitrile containing 0.1% formic acid (B). The mobile phase B was first set at 5% and raised incrementally to 90% in 16 min. It then remained at 90% for 4 min, before returning to the baseline condition (5% B) for the final 25 min. The injection volume was 3 l, and the column temperature was adjusted to 40 °C. The untargeted screening was performed at either positive or negative ionization polarity/state using the full MS/dd-MS2 acquisition mode. Sheath, auxiliary, and sweep gases all contained nitrogen and had arbitrary unit settings of 32, 8, and 4, respectively. The capillary temperature was set at 320 kV, the spray voltage was 3.30 kV, and the auxiliary gas heater temperature was 30 kV. The resolution in both positive and negative ionization modes was 70,000 for full MS and 17,500 for dd-MS2, and the scan range was 66.7–1,000 m/z. Thermo Scientific, Bremen, Germany’s XCalibur 4.4 software was used to control the machine. The instrument was tuned and calibrated once a week in both ESI positive and negative modes using Thermo Scientific Pierce ESI ion calibration solutions (Waltham, MA) to ensure optimal and robust important performances throughout the analysis in terms of mass accuracy (5 ppm), ion transfer, ion isolation, and instrumental sensitivity.

### Chemometric analysis

The metabolites identified from the TIC of both silage and rumen samples (silage and ruminal fluid with and without chitosan inclusion) were used as variables for partial least squares-discriminant analysis (PLS-DA) and principal component analysis (PCA). SIMCA software was utilized to conduct the chemometrics analysis. The metabolites/compounds identified by untargeted analysis served as variables for the PCA and PLS-DA analyses. Twelve samples, consisting of SM1 (silage without chitosan inclusion in the 50% water MS grade: 50% MeOH MS grade solvent); SM2 (silage without chitosan inclusion in the 20% water MS grade: 80% MeOH MS grade solvent); SM3 (silage without chitosan inclusion in the 0% water MS grade: 100% MeOH MS grade solvent); RM1 (ruminal fluid without chitosan inclusion in the 50% water MS grade: 50% MeOH MS grade solvent); RM2 (ruminal fluid without chitosan inclusion in the 20% water MS grade: 80% MeOH MS grade solvent); RM3 (ruminal fluid without chitosan inclusion in the 0% water MS grade: 100% MeOH MS grade solvent); SC1 (silage with 0.5% chitosan inclusion in the 50% water MS grade: 50% MeOH MS grade solvent); SC2 (silage with 0.5% chitosan inclusion in the 20% water MS grade: 80% MeOH MS grade solvent); SC3 (silage with 0.5% chitosan inclusion in the 0% water MS grade: 100% MeOH MS grade solvent); RSC1 (ruminal fluid with 0.5% chitosan inclusion in the 50% water MS grade: 50% MeOH MS grade solvent); RSC2 (ruminal fluid with 0.5% chitosan inclusion in the 20% water MS grade: 80% MeOH MS grade solvent); and RSC3 (ruminal fluid with 0.5% chitosan inclusion in the 0% water MS grade: 100% MeOH MS grade solvent), were used for chemometrics analysis. The variables were scaled using the Pareto scaling technique before being used for creating PCA and PLS-DA models, both for rumen and silage samples. The *R*² and *Q*² values from the PCA were used to evaluate the model.

Meanwhile, the PLS-DA was evaluated using the values of R2X, R2Y, and Q2. Variable importance for projection (VIP) value was used to identify potential biomarkers to discriminate among samples, both in silage and rumen. The metabolite with a VIP value > 1.0 was selected as the discriminating metabolite. According to Artegoitia et al. [9], values >1.0 for a variable in the projection indicate that the metabolite is strongly engaged in the division of groups. In addition, cross-validation was used to validate the discrimination model of PLS-DA and the receiver operating characteristics. The metabolites were annotated using the Kyoto Encyclopedia of Genes and Genomes (KEGG), Human Metabolome Database (HMDB), and PubChem databases.

## Results and Discussion

### Silage samples

The Compound Discoverer software tool was used to identify approximately 200 metabolite characteristics from a silage sample. Amino acids, organic acids, amines, fatty acids, flavones, and organic compounds are among these. The number of extracted metabolites differed depending on the water/MeOH ratio. In the 100% MeOH extract, 246 compounds were detected using the positive and negative ion modes, while 246 compounds were found in the MeOH:Water 80:20 extract and 208 compounds were found in the MeOH:Water 50:50 extract observed on silage with and without chitosan addition. Certain compounds were found in both the MeOH and water extracts. The small number of metabolites from MeOH extraction could be because MeOH is not polar enough to fully extract the extremely polar lipid species [18]. For reconstitution and extraction of biological samples, maximum coverage will be achieved by combining an organic and an aqueous solvent, which strikes a balance between hydrophobicity and hydrophilicity [19]. Even though MeOH/ACN/H₂O generally produces a small number of metabolites across the limit of detection (LOD), it may be a viable choice for this particular combination of sample type and metabolite class. Furthermore, in addition to the distinct benefits and drawbacks of every method for producing adequate numbers of metabolites beyond the LOD, it may be necessary to examine the complexity of the protocol and the availability of the chemical components required by the various protocols [18]. In addition, the extraction solvent MeOH is used for both quenching and extraction of metabolites, which is fast and can retrieve a broad range of metabolites [20]. In terms of extractability and repeatability, MeOH was preferred [13].

**Figure 1.**
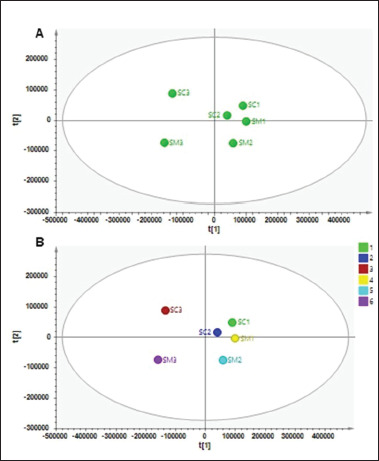
Results of PCA (A) and partial least square-discriminant analysis (B) to differentiate silage samples (SM = silage sample without chitosan, SC = silage sample with chitosan inclusion, 1 = 50% MeOH:50% water, 2 = 80% MeOH:20% water, 3 = 100% MeOH). *SM1 (silage without chitosan inclusion in the* 50% water MS grade: 50% MeOH MS grade solvent)*, SM2 (silage without chitosan inclusion in the* 20% water MS grade: 80% MeOH MS grade solvent)*, SM3 (silage without chitosan inclusion in the* 0% water MS grade: 100% MeOH MS grade solvent)*, RM1 (ruminal fluid without chitosan inclusion in the* 50% water MS grade: 50% MeOH MS grade solvent)*, RM2 (ruminal fluid without chitosan inclusion in the* 20% water MS grade: 80% MeOH MS grade solvent)*, RM3 (ruminal fluid without chitosan inclusion in the* 0% water MS grade: 100% MeOH MS grade solvent)*, SC1 (silage with 0.5% chitosan inclusion in the* 50% water MS grade: 50% MeOH MS grade solvent)*, SC2 (silage with 0.5% chitosan inclusion in the* 20% water MS grade: 80% MeOH MS grade solvent)*, SC3 (silage with 0.5% chitosan inclusion in the* 0% water MS grade: 100% MeOH MS grade solvent)*, RSC1 (ruminal fluid with 0.5% chitosan inclusion in the* 50% water MS grade: 50% MeOH MS grade solvent)*, RSC2 (ruminal fluid with 0.5% chitosan inclusion in the* 20% water MS grade: 80% MeOH MS grade solvent)*, and RSC3 (ruminal fluid with 0.5% chitosan inclusion in the* 0% water MS grade: 100% MeOH MS grade solvent).

The results of PCA using four principal components (PCs) with a total variance of 97% could be used to differentiate silage samples (*R*² = 0.970, *Q*² = 0.588), as illustrated in Figure 1A. Using the first two PCs (PC1 = 59.5%, PC2 = 19.3%), silage samples of SC1, SM1, SC2, and SM2 were grouped into the same cluster. Because PCA is an instrument for unsupervised pattern identification, it is able to reduce data dimensionality and reveal the underlying variation within the data. In the PCA scatter plot, similar datasets are grouped closer together, whereas diverse datasets are placed farther apart [21]. Meanwhile, silage samples of SC3 and SM3 appeared in different clusters separately, indicating differences in the metabolites contained in SC3 and SM3 compared to other samples. This result is according to a study from Fonseca et al. [5], in which PCA scores from the MeOH method are assigned to a distinct area from the ACN/MeOH procedure. This indicated that the use of 100% MeOH as the extraction solvent for silage samples, both with and without the inclusion of chitosan, affected the extracted metabolites from ruminal samples. The 100% MS grade MeOH was utilized as the extraction solvent for silage samples because previous research indicated that it is the preferred solvent for metabolomic analysis because of its broad coverage of semi-polar and polar metabolites [2].

In line with the differentiation result from PCA, the PLS-DA score plot showed similar results with PCA (Fig. 1B). Using five latent variables, PLS-DA demonstrated good fitness and good predictivity with the values of R²X, R²Y, and Q² above 0.990. PLS-DA, a supervised technique for pattern identification, combines PLS-DA algorithms [2].

VIP analysis identified the discriminating metabolites (VIP > 1.0) to discriminate each silage sample, as shown in Table 1. The validity of the PLS-DA model was confirmed using cross-validation and receiver operating characteristic tests. Table 1 presents the important metabolites identified in the VIP values, which were used to divide the silages using the various extraction methods and solvents. VIP scores >1 were present in 60 metabolites in total, which can be used to distinguish metabolites. Potential metabolite markers included organic acids, amino acids, amines, fatty acids, and flavones, among other substances. Cinnamic acid has the greatest VIP value of a putative metabolite marker, followed by D-(+)-proline, L(+)-ornithine, L-valine, and genistein. Acrylic acid with a phenyl group at position 3 makes up cinnamic acid, an organic monocarboxylic acid. These organic aromatic compounds belong to benzene and a carboxylic acid group to generate 3-phenylprop-2-enoic acid. Furthermore, cinnamic acid is a polyphenol that is among the most prevalent plant secondary metabolites (e.g., cinnamon, grapes). Cinnamic acid and its derivatives possess a variety of biological actions, including anti-inflammatory, antiviral, antibacterial, antifungal, antioxidant, and anticancer effects [22]. Cinnamic acid has been linked to lactic acid bacteria, specifically *Latilactobacillus sakei*, which have anti-inflammatory and antibacterial properties [23]. This hypothetical situation could explain why cinnamic acid had the highest VIP score of any metabolite discovered in silage samples. D-(+)-proline, L(+)-ornithine, and L-valine are members of the amino acid compound that have a high VIP value in the silage sample. One isomer of the amino acid found naturally is called D-proline. L-proline is one of the class of chemical compounds known as proline and derivatives. Ornithine or L-ornithine, also known as (S)-2,5-diaminopentanoic acid, belongs to the L-alpha-amino acid class of substances. It is formed during the urea cycle by the separation of urea from arginine. L-Ornithine eliminates excess nitrogen and serves as a precursor for citrulline and arginine. It is classified as a non-essential amino acid by the. L-Valine is an aliphatic and very hydrophobic necessary amino acid in humans that is associated with leucine. Valine is present in several proteins, mostly inside globular proteins, where it aids in the determination of the three-dimensional form. Soy, seafood, cheese, meats, and vegetables contain valine. L-Valine is involved in protein digestion and absorption as a precursor for enzymes and hormones, particularly growth hormone, metabolic pathways, secondary metabolite biosynthesis, leucine, valine, and isoleucine degradation-biosynthesis, and amino acid biosynthesis. Amino acids have been reported as metabolites in silage metabolomic studies. This finding is congruent with the findings of [6,24], who discovered an amino acid compound in treated silage with various additives in their investigation. As a result of glycolysis, fatty acid metabolism, and proteolysis, the primary microbial metabolic pathways that determine the taste and quality of silage are carbohydrate and amino acid metabolisms. Amino acids are mostly metabolites produced by bacteria through metabolic processes. They are essential chemicals in plants and play major functions in plant protein synthesis and primary metabolism [23].

**Table 1.** Discriminating metabolites obtained from VIP value analysis (VIP >1.0) to discriminate silage samples.

No. Metabolites	VIP value	Molecular formula	Calculated m/z	Retention time (min)
1 Cinnamic acid	5.62	C_5_H_9_NO_2_	115.06304	1.054
2 D-(+)-Proline	4.23	C_5_H_12_N_2_O_2_	132.08958	1.045
3 L(+)-Ornithine	4.23	C_5_H_11_NO_2_	117.07875	1.258
4 L-Valine	3.84	C_15_H_10_O_5_	270.05197	10.488
5 Genistein	3.80	H_2_O_4_S	97.96783	0.98
6 Sulfuric acid	3.58	C_9_H_11_NO_2_	165.07841	2.67
7 L-Phenylalanine	3.24	C_6_H_13_NO_2_	131.09421	1.365
8 L-Isoleucine	3.10	C_6_H_13_NO_2_	131.09417	1.81
9 L-Norleucine	3.05	C_10_H_18_N_2_O_3_	214.13097	2.147
10 Valylproline	3.01	C_18_H_30_O_2_	278.22341	15.962
11 α-Eleostearic acid	2.79	C_15_H_10_O_4_	254.05717	9.244
12 Daidzein	2.77	C_13_H_11_NO_2_	213.07819	5.991
13 Fenamic Acid	2.69	C_18_H_32_O_4_	312.22971	16.148
14 (±)9-HpODE	2.50	C_12_H_23_NO_7_	293.14655	1.886
15 (2S)-4-Methyl-2-({[(3S,4S,5R)-2,3,4-trihydroxy-5-(hydroxymethyl)tetrahydro-2-furanyl] methyl}amino)pentanoic acid (non-preferred name)	2.35	C_11_H_20_N_2_O_3_	228.14673	3.67
16 Leucylproline	2.29	C_13_H_14_N_2_O_2_	230.10463	6.012
17 1-Methyl-1,2,3,4-tetrahydro-Î²-carboline-3-carboxylic acid	2.24	C_5_H_8_O_2_S	132.02406	1.284
18 3-Methylsulfolene	2.18	C_5_H_11_NO_2_S	149.05062	1.262
19 L-(-)-Methionine	2.12	C_10_H_16_N_2_O_4_	228.11027	2.596
20 Tetraacetylethylenediamine	2.02	C_6_H_14_N_2_O_2_	146.10504	1.038
21 DL-Lysine	1.92	C_4_H_5_N_3_O	111.04306	1.139
22 Cytosine	1.89	C_18_H_39_NO_3_	317.29153	12.507
23 Phytosphingosine	1.83	C_6_H_11_NO_3_	145.07333	1.211
24 2-morpholinoacetic acid	1.83	C_6_H_15_O_4_P	182.0701	8.805
25 Triethyl phosphate	1.72	C_15_H_21_NO_7_	327.13069	2.818
26 (2S)-3-Phenyl-2-({[(3S,4S,5R)-2,3,4-trihydroxy-5-(hydroxymethyl)tetrahydro-2-furanyl] methyl}amino)propanoic acid (non-preferred name)	1.70	C_24_H_38_O_4_	390.27541	20.22
27 Di(2-ethylhexyl) phthalate	1.64	C_14_H_15_N	197.11978	6.855
28 Dibenzylamine	1.63	C_7_H_14_N_2_O_3_	174.10002	1.231
29 L-Theanine	1.62	C_18_H_32_O_3_	296.23396	17.393
30 13S-hydroxyoctadecadienoic acid	1.60	C_18_H_34_O_2_	282.25487	17.706
31 Ethyl palmitoleate	1.59	C_12_H_24_N_2_O_3_	244.17771	5.479
32 Leu-Leu	1.56	C_18_H_30_O_2_	278.22342	15.579
33 α-Linolenic acid	1.54	C_20_H_34_O_2_	306.25461	19.022
34 Linolenic acid ethyl ester	1.49	C_10_H_20_N_2_O_3_	216.14658	2.037
35 Valylvaline	1.47	C_16_H_12_O_5_	284.06764	9.483
36 Glycitein	1.47	C_23_H_44_NO_7_P	477.28424	14.935
37 1-linoleoyl-sn-glycero-3-phosphoethanolamine	1.46	C_5_H_7_NO_3_	129.04215	1.301
38 L-Pyroglutamic acid	1.39	C_18_H_30_O_3_	294.21828	16.369
39 13(S)-HOTrE	1.38	C_11_H_20_N_2_O_5_	260.13646	1.736
40 L-gamma-Glutamyl-L-leucine	1.38	C_18_H_28_O_3_	292.20279	15.144
41 12-oxo Phytodienoic Acid	1.33	C_14_H_19_NO_6_	297.12042	3.364
42 N-(2-Phenylethyl)-beta-D-glucopyranuronosylamine	1.33	C_10_H_14_N_2_O_4_	226.09474	3.266
43 Porphobilinogen	1.32	C_11_H_22_N_2_O_3_	230.16226	4.443
44 Leu-Val	1.30	C_9_H_18_N_2_O_4_	218.12586	2.589
45 Meprobamate	1.29	C_5_H_4_N_4_O	136.0381	1.307
46 Hypoxanthine	1.28	C_11_H_9_NO_2_	187.06272	4.57
47 trans-3-Indoleacrylic acid	1.25	C_18_H_39_NO_3_	317.29147	11.24
48 2-Amino-1,3,4-octadecanetriol	1.20	C_18_H_34_O_5_	330.24027	10.952
49 (15Z)-9,12,13-Trihydroxy-15-octadecenoic acid	1.12	C_10_H_9_NO_4_	207.05231	5.747
50 4-(2-Aminophenyl)-2,4-dioxobutanoic acid	1.10	C_12_H_21_NO_6_	275.13603	1.869
51 Glutarylcarnitine	1.09	C_8_H_8_	104.06248	3.255
52 Styrene	1.09	C_18_H_32_O	264.24445	17.707
53 2-[(5Z)-5-tetradecenyl]cyclobutanone	1.07	C_6_H_12_O_7_	196.05754	1.194
54 Gluconic acid	1.06	C_14_H_18_N_2_O_3_	262.13104	4.838
55 Methohexital	1.06	C_11_H_21_NO_7_	279.13094	1.274
56 (2S)-3-Methyl-2-({[(3S,4S,5R)-2,3,4-trihydroxy-5-(hydroxymethyl)tetrahydro-2-furanyl] methyl}amino)butanoic acid (non-preferred name)	1.04	C_18_H_34_O_4_	314.24486	14.195
57 (+/-)9,10-dihydroxy-12Z-octadecenoic acid	1.02	C_10_H_20_N_2_O_4_	232.14157	2.901
58 Mebutamate	1.01	C_18_H_30_O_4_	310.21362	15.266
59 13(S)-HpOTrE	1.01	C_4_H_11_O_4_P	154.03895	8.806
60 Diethyl phosphate	1.01	C_11_H_22_N_2_O_3_	230.16227	3.119

**Figure 2.**
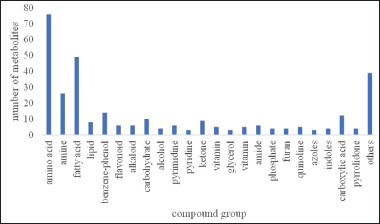
Compound group of the total mixed ration silage.

In this investigation, a total of 246 metabolites were discovered with the use of 100% MeOH for the solvent extraction. To comprehend the functional properties and categorizations of various metabolites, the discovered metabolites were annotated in the National Center for Biotechnology Information (NCBI)-PubChem database, HMDB database, and KEGG database. The analysis revealed that the amino acid category contained 76 metabolites, surpassing other categories. Additionally, there were 49 metabolites in the fatty acid compounds category and 26 in the amine compound category. The remaining metabolites are shown in Figure 2.

**Figure 3.**
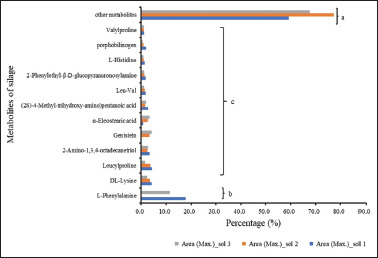
Percentage metabolite area of silage sample due to different solvents.

Organic compounds are a diverse set of chemicals in which one or more carbon atoms are covalently bonded to atoms of other elements, mostly oxygen, hydrogen, or nitrogen. The superclass consisted primarily of peptides (40.2% oligopeptides and 8.4% dipeptides) and amino acids (8.6%), according to (24). Furthermore, the utilization of metabolomic profiling is a highly valuable approach for comprehensively examining the fermentative, nutritional, and functional characteristics of ensiled forages intended for animal consumption [6]. Nevertheless, 123 metabolites were found to be common in the silage samples in all solvents used. Figure 3 shows that L-phenylalanine had the highest area metabolite (*p* < 0.05) among other common metabolites. L-Phenylalanine is a crucial amino acid used as an intermediate in the synthesis of various biological compounds [25,26]. It is frequently utilized in the pharmaceutical, food, and chemical industries, particularly in the manufacturing of aspartame (a popular sweetener) and various drugs with antiviral and anticancer properties [27]. This compound is effectively extracted using solvents 1 and 3, as indicated by the high metabolite area. This outcome aligns with the solubility of L-phenylalanine in water, MeOH-water, and ethanol–water mixtures within the temperature range of 288.15–318.15 K, utilizing its anhydrous form [27]. Furthermore, water was a more potent solvent for l-phenylalanine than other solvents. MeOH may be utilized as a beneficial anti-solvent in the crystallization process [27].

### Ruminal fluid samples

The software application Compound Discoverer was utilized to detect around 20–40 metabolite characteristics from ruminal fluids. Among them are amines, organic phosphates, fatty acids, and organic compounds. The number of extracted metabolites varied with the water/MeOH ratio. The positive and negative ion modes detected 43 compounds in the 100% MeOH extract, 31 compounds in the MeOH:Water 80:20 extract, and 38 compounds in the MeOH:Water 50:50 extract observed on ruminal fluids with and without chitosan addition. Fifty percent H₂O in MeOH, a combination of organic and aqueous solvents, appears to be an increasingly common option. This could be a method for widening metabolome coverage by including hydrophilic and hydrophobic compounds that are poorly soluble in pure water [19]. When a MeOH/water combination was used to extract intracellular metabolites, it was discovered that the volume ratio of MeOH to water solvent influenced the extraction efficacy [14].

**Figure 4.**
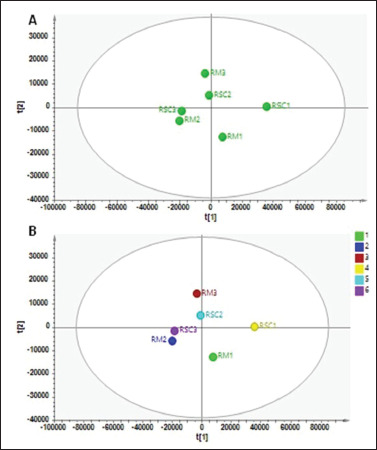
The score plot of PCA (A) and partial least square-discriminant analysis (PLS-DA) (B) of different ruminal fluid samples (RM = ruminal fluid without chitosan, RSC = ruminal fluid with chitosan inclusion, 1 = 50% MeOH:50% water, 2 = 80% MeOH:20% water, 3 = 100% MeOH), *SM1 (silage without chitosan inclusion in the* 50% water MS grade: 50% MeOH MS grade solvent)*, SM2 (silage without chitosan inclusion in the* 20% water MS grade: 80% MeOH MS grade solvent)*, SM3 (silage without chitosan inclusion in the* 0% water MS grade: 100% MeOH MS grade solvent)*, RM1 (ruminal fluid without chitosan inclusion in the* 50% water MS grade: 50% MeOH MS grade solvent)*, RM2 (ruminal fluid without chitosan inclusion in the* 20% water MS grade: 80% MeOH MS grade solvent)*, RM3 (ruminal fluid without chitosan inclusion in the* 0% water MS grade: 100% MeOH MS grade solvent)*, SC1 (silage with 0.5% chitosan inclusion in the* 50% water MS grade: 50% MeOH MS grade solvent)*, SC2 (silage with 0.5% chitosan inclusion in the 20*% water MS grade: 80% MeOH MS grade solvent)*, SC3 (silage with 0.5% chitosan inclusion in the* 0% water MS grade: 100% MeOH MS grade solvent)*, RSC1 (ruminal fluid with 0.5% chitosan inclusion in the* 50% water MS grade: 50% MeOH MS grade solvent)*, RSC2 (ruminal fluid with 0.5% chitosan inclusion in the* 20% water MS grade: 80% MeOH MS grade solvent)*, and RSC3 (ruminal fluid with 0.5% chitosan inclusion in the* 0% water MS grade: 100% MeOH MS grade solvent).

On the other hand, unsupervised pattern recognition using PCA along with supervised pattern recognition of PLS-DA was applied for the differentiation of ruminal samples with and without the inclusion of chitosan extracted using three different extraction solvents (Fig. 4). The PCA was built using four PCs, resulting in a total variance of 98.2% with *R*² = 0.982 and *Q*² = 0.811. The PCA score plot (Fig. 4A) using PC1 and PC2 (PC1 = 67.7%, PC2 = 14%) showed the pattern of sample grouping based on their metabolite compositions. Samples of RM2 and RSC3 were tightly clustered in the same area, whereas samples of RM3 and RSC2 appeared close to each other. These indicated the similarity of the metabolite compositions between RM2-RSC3 and RM3-RSC2, respectively. On the other hand, samples of RM1 and RSC1 appeared in a different cluster, with RSC1 having the largest distance to all rumen samples. It is associated with the different metabolite compositions of RSC1 compared to others, indicating that the use of 50% water: 50% MeOH as the extraction solvent applied to ruminal fluid samples containing chitosan resulted in different metabolite compositions compared to others. This study utilized a solvent mixture consisting of 50% MS-grade MeOH and 50% MS-grade water to extract ruminal fluid samples. This choice was based on prior research that demonstrated the effectiveness of this solvent in extracting ruminal fluid [9,10]. Increasing metabolome coverage (30%–50%) by using aqueous rather than organic conditions for the metabolite extraction step [19].

Because of the changes in solvent polarity, the solvent used for extraction alters the overall appearance of the separated bioactive chemicals present in the extract [28]. The total metabolites and the extraction method utilized were heavily influenced by the type of solvent used [4]. The observed variations between solvents were mostly due to differences in their properties, primarily polarity, selectivity, toxicity, and inertness, and hence the solubility of particular chemicals in the extraction solvents [14]. Apart from PCA, the score plot result of PLS-DA (Fig. 4B) demonstrated the discrimination results of different ruminal fluid samples. The sample grouping was in accordance with the results from the PCA analysis. The PLS-DA was created using five latent variables, resulting in R²X, R²Y, and Q² values more than 0.990, showing good fitness and good predictive ability of the model. Using PLS-DA, the discriminating metabolites important for VIP values more than 1.0 are considered to have important roles in the discrimination of ruminal fluid samples, as depicted in Table 2. The validity of the PLS-DA model was assessed using a cross-validation test and receiver operating characteristics.

Table 2 presents the important metabolites found in the VIP values, which are useful for differentiating ruminal fluids with and without the addition of chitosan. Eleven metabolites with VIP scores greater than one can be used as metabolite markers. Triethyl phosphate (TEP) had the greatest VIP value for any possible metabolite marker, followed by dibenzylamine and phosphoric acid. TEP or ethyl phosphoric acid is an organic compound that belongs to the trialkyl phosphate class. TEP is the triethyl ester derivative of phosphoric acid and is functionally related to ethanol. Dibenzylamine belongs to the class of chemical compounds known as phenylmethylamine (amine compounds).

**Table 2.** Discriminating metabolites obtained from VIP value analysis (VIP >1.0) to discriminate ruminal fluid samples

No.	Metabolites	VIP value	Molecular formula	Calculated m/z	Retention time (min)
1	Triethyl phosphate	2.59	C_6_H_15_O_4_P	182.07006	8.841
2	Dibenzylamine	2.30	C_14_H_15_N	197.11962	6.976
3	Phosphoric acid	1.74	H_3_O_4_P	97.97668	1.473
4	Sphinganine	1.61	C_18_H_39_NO_2_	301.29699	12.521
5	Diethyl phosphate	1.62	C_4_H_11_O_4_P	154.03889	8.839
6	2,2'-Methylenebis(4-methyl-6-tert-butylphenol)	1.45	C_23_H_32_O_2_	340.23978	19.379
7	Bis(4-ethylbenzylidene)sorbitol	1.39	C_24_H_30_O_6_	414.20262	13.984
8	Capsi-amide	1.38	C_17_H_35_NO	269.27097	20.808
9	Lauramide	1.27	C_12_H_25_NO	199.19294	15.616
10	Navenone A	1.24	C_15_H_15_NO	225.11463	12.995
11	1-(14-methylhexadecanoyl)pyrrolidine	1.05	C_21_H_41_NO	323.31753	22.702

Furthermore, amines have been implicated in certain processes, such as betalain production, adipocyte lipolysis control, methane metabolism, protein digestion and absorption, and antifungal agents. Ruminants, on the other hand, can receive biogenic amines derived from both food and rumen microbial metabolites, and biogenic amines are regularly produced through the decarboxylation of certain amino acids. Rumen microbial bacteria boost amino acid metabolism, which may be aided by a lower rumen pH [29]. Phosphoric acid is a phosphorus oxoacid composed of one oxo and three hydroxy groups covalently linked to a central phosphorus atom. It is a solvent, a human metabolite, and an algae metabolite. Organic phosphates play vital roles in biology, biogeochemistry, and ecology. Phosphates are most typically found in DNA and RNA as adenosine phosphates (AMP, ADP, and ATP) and can be released via the hydrolysis of ATP or ADP.

**Figure 5.**
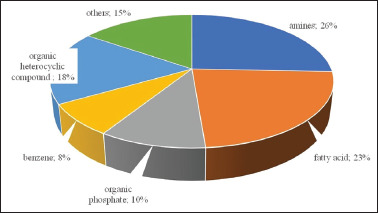
Compound group of ruminal fluid metabolite samples.

In addition, a comprehensive total of 38 metabolites were found throughout the course of this study. In order to comprehend the functional attributes and categorizations of various metabolites, the discovered metabolites were annotated in the NCBI-PubChem database, HMDB database, and KEGG database. The analysis revealed that amine compounds were the most prevalent, with 10 discovered metabolites. Fatty acids were the second most common, with nine detected metabolites. Organic heterocyclic compounds accounted for seven metabolites, and the remaining metabolites are shown in Figure 5. This finding aligns with the research conducted by Yang et al. [21], which indicates that phospholipids, inorganic ions and gases, dicarboxylic acids, amino acids, short-chain fatty acids, diglycerides, triglycerides, glucose, carbohydrate cholesterol esters, organic acids, two peptides, and lipids are the predominant components in bovine ruminal fluid. Several of these substances are the result of microbial fermentation occurring in the anaerobic environment of the rumen [9].

**Figure 6.**
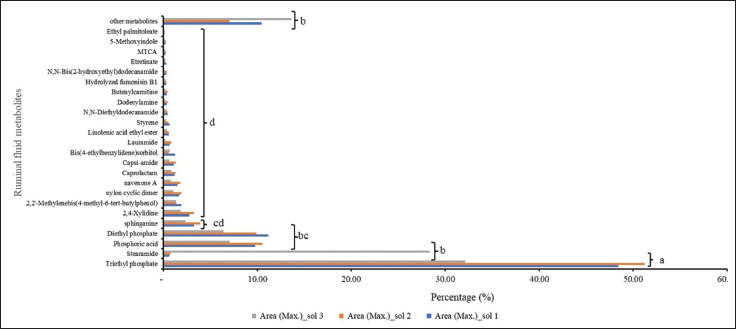
Percentage metabolite area of the ruminal fluid sample due to different solvents.

Metabolomics allows the identification of a network of biological markers that indicate physiological and pathological processes. This allowed us to highlight the phenotypic changes observed between distinct groups of animals [30]. The utilization of metabolomics in ruminant research has facilitated the identification of several chemical substances present in biological tissues or fluids [31]. The assessment of overall metabolism and biology has progressed. These metabolites are generated by several enzymatic mechanisms and metabolic pathways [30]. Nevertheless, 25 metabolites were found to be common in all solvents used in ruminal fluid samples. Figure 6 shows that TEP had the highest area metabolite, followed by stearamide (*p* < 0.05), among other common metabolites. TEP is well extracted in solvents 1 and 2 based on the high metabolite area of this compound, which is very soluble in organic solvents [19]. Stearamide, also known as octadecanamide, is a fatty acid of stearic acid. It also acts as a metabolite. It is an organic compound found in the *Bos taurus*. Steramides are included in lipid compounds, especially fatty amide compounds [32]. Another fact is that this metabolite is well extracted in solvent three, which consists of 100% MeOH. This is because this compound is included in lipid compounds, so it is insoluble in water but slightly soluble in MeOH compounds [32].

However, despite the advantages of this research, the limitations of the study regarding the metabolomic profiles of silage digested in ruminal fermentation with various solvent extraction techniques mainly stem from potential biases introduced by the extraction methods, the complexity of metabolomic data, and the relevance of *in vitro* findings to *in vivo* conditions. The choice of solvent in the extraction process can significantly influence which metabolites are detected and quantified. Different solvents may extract metabolites with varying efficiencies, potentially leading to incomplete or biased metabolomic profiles. In addition, metabolomic data are often high-dimensional and complex, involving many metabolites with diverse chemical properties. Therefore, appropriate statistical analysis is required to avoid misleading conclusions about which metabolites are significantly affected by ruminal fermentation or solvent extraction. To strengthen the study’s conclusions, future research could address these limitations by incorporating larger sample sizes, more comprehensive solvent extraction techniques, and broader metabolic pathway analyses while ensuring that results are translatable to real-world ruminal fermentation in animals.

## Conclusion

The TMR silage samples contained 311 compounds. The most prevalent ones were amino acid compounds, followed by fatty acids and amines. The 39 metabolites found in the rumen fluid samples were mostly composed of fatty acids, organic phosphates, and amines. The PCA results show that different solvent amounts lead to different outcomes. In the TMR silages, 100% MeOH seemed to be better at separating samples from other solvents. In contrast, for rumen fluid samples, the best results were obtained using a liquid combination of 50% MeOH and 50% water.

## List of abbreviations

ADP, Adenosine Diphosphate

AMP, Adenosine Monophosphate

ATP, Adenosine Triphosphate

CAN, Acetonitrile

ESI, Electrospray Ionization

HMDB, Human Metabolome Database

KEGG, Kyoto Encyclopedia of Genes and Genomes

LC-HRMS, liquid chromatography-high resolution mass spectrometry

LOD, limit of detection

MeOH, methanol

MS, Mass Spectrometry

NCBI, National Center for Biotechnology Information

PCA, principal component analysis

PCs, principal components

PLS-DA, partial least square-discriminant analysis

PTFE, Polytetrafluoroethylene

RC, rumen fluid containing 0.5%

chitosan

RM, ruminal fluid without chitosan

RSC, ruminal fluid with chitosan

SC, silage with 0.5%

inclusion of chitosan

SM, silage without chitosan addition

TEP, triethyl phosphate

TMR, total mixed ration

TIC, Total Ion Chromatogram

UHPLC, Ultra-High Performance Liquid Chromatography

VIP, variable importance for projection.
